# The Role of Lecturer–Student Interaction in Developing Critical Thinking Skills: Insights from a Private University in Indonesia

**DOI:** 10.12688/f1000research.173679.1

**Published:** 2025-12-26

**Authors:** Teguh Prasetyo, Helmia Tasti Adri, Fachri Helmanto

**Affiliations:** 1 Faculty of Teacher Training and Education, Djuanda University, Bogor, West Java, 16720, Indonesia; 2 Faculty of Teacher Training and Education, Djuanda University, Bogor, West Java, 16720, Indonesia; 3 Faculty of Teacher Training and Education, Universitas Djuanda, Bogor, West Java, 16720, Indonesia

**Keywords:** Lecturer–Student Interaction, Critical Thinking Skills, Student Engagement, Classroom Dialogue, Higher Education

## Abstract

**Background:**

The development of critical thinking remains a challenge in private universities (PTS) in Indonesia. Learning interactions in PTS are often influenced by interpersonal closeness, cultural norms, and communication patterns that do not always support the courage to express opinions. Although previous research has emphasized the roles of lecturer-student interactions and student collaboration, empirical studies explaining how the form and quality of these interactions influence critical thinking are limited. This study aims to describe the forms of interaction, the quality of pedagogical support, and the barriers that influence the development of students’ critical thinking.

**Methods:**

This qualitative study involved 12 students from a private university in Bogor. Data were collected through semi-structured interviews and analyzed thematically to identify patterns of lecturer-student interactions, peer collaboration, and cultural factors that influence participation.

**Results:**

The results indicate that supportive lecturer-student interactions—including constructive feedback, positive emotional responses, and warm communication—increase students’ confidence and engagement in critical thinking activities. Peer collaboration helps broaden perspectives and cultivate higher-order reasoning. However, obstacles persist, including uneven lecturer support, unequal group contributions, and students’ tendency to remain silent due to hierarchy, politeness norms, and fear of judgment.

**Conclusions:**

Positive and culturally sensitive learning interactions play a crucial role in fostering critical thinking in private universities. Consistent pedagogical support and structured collaboration are necessary to foster an inclusive learning environment for critical thinking.

## Introduction

Lecturer–student interaction is widely recognized as one of the most influential factors shaping students’ learning experiences and academic development. Positive, supportive, and dialogic interactions have been shown to enhance students’ engagement, comfort, and satisfaction with the learning process ( Che Ahmad et al., 2017). Such interactions create an environment where students feel encouraged to participate actively, express ideas, and build confidence—elements essential to deeper learning and the development of critical thinking. Evidence from Indonesian higher education further highlights the unique interactional characteristics of private universities. Wulandari and Jager (2018) reported that private institutions tend to adopt more customer-oriented approaches, leading students to expect more accessible lecturers, more transparent communication, and more individualized academic guidance. Because private universities generally operate with smaller class sizes and more flexible administrative structures, students often experience closer and more personal interaction with lecturers. Such conditions make private universities an important setting for examining how lecturer–student interaction contributes to students’ cognitive and critical thinking development.

From a social constructivist perspective, learning is fundamentally mediated through social interaction. Vygotsky argues that knowledge is first constructed through collaborative dialogue before being internalized as higher-order thinking ( Wan Hussin et al., 2019a). In this sense, lecturer–student interaction serves as scaffolding that guides students to analyze, evaluate, and reflect more deeply. However, research suggests that differences in lecturers’ beliefs about critical thinking—whether it is viewed as a teachable skill or a fixed ability—can affect the extent to which classroom interactions truly support students’ cognitive development ( Indrašienė et al., 2023).

However, how students participate in social interaction is powerfully shaped by cultural norms. Chung (2021), studying Thai undergraduates, showed that students often engage through “silent participation”—a form of attentive, reflective engagement expressed through deliberate quietness. Rather than indicating passivity, silence is used to maintain social harmony and avoid the risk of negative judgment. This pattern, typical across Southeast Asian learning cultures, has important implications for how students negotiate dialogue, express ideas, and engage in higher-order thinking during classroom interaction.

Similar findings have been observed in other Southeast Asian contexts. Luu (2022) showed that academic interactions in high-power-distance cultures—such as Vietnam—tend to foster cautious, deliberate communication. Students often engage in polite and non-confrontational forms of participation, especially when taught by lecturers from different cultural backgrounds, such as in English-Medium Instruction (EMI) contexts. Variations in lecturers’ teaching styles and local cultural norms lead students to prefer listening over questioning or arguing. These findings confirm that Asian academic culture plays a significant role in how students interact and develop critical thinking skills.

Previous research highlights that lecturers’ active teaching behaviours—such as providing guidance, asking probing questions, and encouraging participation—significantly enhance student engagement and satisfaction. These forms of pedagogical interaction help establish a learning atmosphere where students feel supported and motivated to contribute, which is essential for the development of higher-order thinking skills. Studies also show that how lecturers structure interactions during learning activities can influence students’ cognitive outcomes, including their critical thinking abilities ( Li et al., 2023).

From a social constructivist perspective, learning develops through social interaction, where dialogue and collaboration guide students toward higher-order thinking ( Wan Hussin et al., 2019a). In this sense, lecturer–student interaction functions as a scaffold that helps students analyze, evaluate, and reflect more deeply. However, differences in lecturers’ beliefs about critical thinking—as either a skill that can be developed or a fixed ability—may influence how effectively such interactions support cognitive growth ( Indrašienė et al., 2023). Therefore, explicit and systematic instructional approaches remain essential for strengthening students’ critical thinking ( Wan & Cheng, 2019).

Understanding the pedagogical and social elements that contribute to students’ critical thinking is therefore essential. Prior studies suggest that active teaching strategies and meaningful academic interactions can enhance students’ responsibility for learning and foster higher-order thinking ( Lu, 2021). These findings highlight the importance of examining how lecturer–student interaction functions as a critical component of the learning process, particularly in higher education contexts.

A similar phenomenon was also found in the Indonesian context. Wijayanti et al. (2023) revealed that many students chose not to ask questions directly to lecturers due to fear of making mistakes, fear of being perceived as incompetent, and feeling awkward speaking in front of their peers. The habit of relying more on written messages during online learning also reinforces this tendency, leading students to engage less frequently in face-to-face dialogue. These findings suggest that students’ reluctance to interact constitutes a communicative barrier that can limit opportunities for critical thinking and for negotiating meaning in learning.

While findings in Southeast Asia Luu (2022) suggest that cultural norms significantly influence interaction patterns, evidence in Indonesia ( Sari et al., 2023) confirms that academic hierarchies also shape how students respond to feedback and participate in classroom dialogue. This situation suggests the need for more in-depth investigation into how lecturer-student interactions contribute to the development of critical thinking skills in the Indonesian context.

Although various instructional models have been shown to influence critical thinking, existing research still provides limited insight into how lecturer–student interaction specifically shapes students’ cognitive development in higher education ( Karapetian, 2020; Lu, 2021). This gap is particularly relevant in the context of private universities in Indonesia, where interactional dynamics may differ from public institutions. Therefore, this study aims to explore how students perceive and experience lecturer–student interactions in relation to the development of their critical thinking skills. Given the central role of interaction in shaping cognitive development, this study investigates how students at a private university in Bogor experience lecturer–student interactions and how these interactions relate to the development of their critical thinking skills. By examining these interactional dynamics, the study contributes to a deeper understanding of the learning processes that support higher-order thinking in higher education. The findings are expected to provide practical insights for lecturers and institutional policymakers in designing more effective and interaction-rich learning environments.

Because lecturer–student interaction is shaped by cultural norms, emotions, and classroom dynamics, a qualitative approach is necessary to capture its depth and complexity. Students’ own experiences provide insights that cannot be obtained through experiments, which tend to force interactions into controlled, unnatural conditions. This case study was chosen because the context of private higher education has distinct interaction characteristics—smaller classes, more intense interpersonal relationships, and more personal communication expectations. By focusing on student experiences, this research seeks to understand how academic interactions are perceived, negotiated, and interpreted as students develop critical thinking skills.

Unlike previous studies that have focused primarily on teaching models or lecturer perspectives, this research offers a novel contribution by centering on students’ lived experiences of interaction within a culturally hierarchical private university context. This perspective provides new insight into how interactional patterns shape the development of critical thinking in settings that remain underexplored in the Southeast Asian higher education literature.

## Methods

### Design

This study employed a qualitative case study design to explore how students experience lecturer–student interaction and learning environment conditions in a private university in Bogor, Indonesia. A qualitative approach was selected because social interaction, students’ perceptions, and the development of critical thinking are context-dependent and cannot be adequately captured by experimental or survey-based methods. A case study enabled an in-depth examination of naturally occurring interactional patterns within a real institutional setting ( Paparini et al., 2020).

### Research subjects

Twelve undergraduate students (7 females, 5 males) across the third, fifth, seventh, and ninth semesters participated in this research. The variation in semester levels and academic backgrounds provided rich, diverse perspectives on learning interactions and the environmental factors influencing critical thinking.

### Data collection

Data were gathered through semi-structured interviews comprising 20 open-ended questions on lecturer–student interaction, classroom communication, and support for the learning environment. Each interview lasted 60–120 minutes and was audio-recorded with prior consent. Two additional participants were involved to validate and cross-check emerging findings. Two additional participants were involved to validate and cross-check emerging findings. Focus group discussions (FGDs) were conducted to triangulate the data and enhance credibility.

### Data analysis

All recordings were transcribed verbatim and analyzed using NVivo 12. Data were coded inductively to identify recurring patterns, categories, and thematic relationships. Member checking was conducted by returning summarized findings to participants to ensure interpretive accuracy.

## Results

This study identified one major themes in students’ learning experiences during lecturer–student interactions aimed at developing critical thinking. The interview data revealed that classroom interaction consisted of both supportive and constraining elements. Most students expressed overall satisfaction with their interactions with lecturers, particularly when lecturers encouraged participation, responded positively to students’ ideas, and facilitated group activities that stimulated deeper engagement.

Students also highlighted that collaborative work and peer discussions created opportunities to exchange ideas and broaden perspectives. These experiences contributed to a learning atmosphere perceived as open, interactive, and academically supportive.

### Positive interactions

Many students reported receiving consistent positive responses from lecturers when expressing their opinions. Such interactions made them feel comfortable sharing ideas and promoted a supportive classroom climate. As one student stated:

• Subject 1: “The interaction with lecturers is supportive … they always give positive responses, and I am always allowed to express my opinion.”
• Subject 2: “The interaction is constructive … lecturers always provide feedback, and I am allowed to share my thoughts.”

These accounts show that students perceived lecturer behaviour as encouraging, responsive, and facilitative in promoting participation.

Most students agreed that positive interactions with lecturers enhanced their learning experience and encouraged them to express ideas more confidently. They described lecturers as responsive, appreciative, and willing to offer students opportunities to contribute during discussions. This recognition made students feel valued and motivated to participate.

When asked about the types of support that promote critical thinking, students highlighted lecturers’ openness to dialogue and their willingness to offer constructive guidance:

• Subject 1: “Lecturers should be like this; for example, giving extra credit to students who share their ideas or opinions.”
• Subject 6: “Lecturers are very open to discussions and guidance. They often encourage us to think critically.”
• Subject 3: “Some lecturers always support me, both academically and non-academically.”

Students also emphasized the importance of lecturers acknowledging their contributions. As one respondent noted, “Their willingness to give feedback on assignments helps us sharpen our analytical and critical skills.” This suggests that appreciation and timely feedback are key elements of positive interaction that contribute to students’ cognitive development.

Another student expressed appreciation for the lecturers’ guidance, noting that this support helped build confidence during class discussions. Students explained that constructive feedback enabled them to refine their analytical and critical thinking skills, making lecturers not only sources of knowledge but also facilitators in their cognitive development. They also found that positive relationships, clear explanations, fair grading, and timely responses from lecturers were associated with higher satisfaction with the learning process.

### Collaboration and group discussion

Students reported that peer interactions during learning played an important role in strengthening their engagement and developing critical thinking. Group discussions were viewed as opportunities to exchange ideas, compare perspectives, and deepen understanding of the material.

Ketika ditanya mengenai pengalaman mereka dengan pembelajaran berbasis kolaborasi, beberapa mahasiswa menyatakan:

• Subject 2: “Interactions with peers can be very positive and supportive. Through tasks and group discussions, we communicate with one another.”
• Subject 4: “My interactions with my friends and lecturers greatly support my development of critical thinking.”

Students also described collaborative activities as empowering, helping them feel more involved in the learning process. One student explained that group discussions allowed them to explore topics more thoroughly and gain new insights from classmates’ viewpoints. These collaborative exchanges were perceived as fostering a supportive classroom atmosphere in which students felt encouraged to participate and share their ideas.

Students explained that group discussions and collaborative tasks helped them exchange ideas more freely and examine the course material from multiple perspectives. They described these interactions as opportunities to clarify concepts, test their understanding, and learn from their peers’ different viewpoints. Several students noted that working in groups increased their confidence because they felt supported by their classmates during discussions.

These collaborative activities also made students feel more engaged in the learning process. Many reported that sharing ideas with peers helped them develop new insights and deepen their understanding of the topics they were studying. Students consistently expressed that collaboration—whether through structured group tasks or informal discussions—created a learning atmosphere in which they felt encouraged to participate and contribute.

### Negative interactions (potential)

**Variations in lecturer support**

Students reported that lecturer support was inconsistent across classes. Some lecturers were described as providing detailed explanations and constructive feedback, while others offered only brief comments or limited opportunities for students to express their ideas. One student stated, “Some lecturers are supportive, but there are also those who do not provide enough opportunities to express opinions.”

Several students explained that receiving unequal levels of guidance made it more difficult for them to understand expectations or improve their performance. They expressed a preference for more precise, specific feedback, particularly when completing assignments or engaging in class discussions. These variations in support contributed to differences in how students experienced learning and how actively they felt able to participate.

**Individual roles in discussions**

Students reported that participation during group discussions was often uneven. Several students noted that a small number of peers tended to dominate the conversation, which limited opportunities for others to contribute. One student expressed, “I feel not everyone has the same opportunity to speak.” Another added, “Some lecturers support, but there are also those who do not give enough chances for us to express our opinions.”

Students also highlighted that they felt more willing to participate when the classroom atmosphere was safe, respectful, and when they were confident that their ideas would be acknowledged. A few students stated that the fear of being dismissed or overshadowed by more vocal peers made them hesitant to speak. These conditions led to unequal involvement and reduced the diversity of perspectives shared during discussions.

**Freedom of speech**

Some students reported feeling constrained when expressing differing or opposing opinions during discussions or presentations. They mentioned concerns about how their comments might be perceived and the possibility of receiving adverse reactions. A student explained that they sometimes chose not to express their views because they were unsure whether their opinions would be valued.

This hesitation made some students more reserved and less inclined to contribute to class dialogue. These responses suggest that some students may experience internal barriers that limit their engagement in open discussion and reduce opportunities for deeper critical thinking.

Although most students described their interactions with lecturers and peers as positive and supportive, the interviews also revealed several challenges that may hinder learning. These included inconsistent lecturer support, unequal participation in discussions, and students’ hesitation to express differing opinions. These findings indicate that, alongside positive interaction patterns, students continue to experience specific interactional barriers in the classroom.

## Discussion

This section should include discussion of the results, with limitations, implications of this study, and recommendations for further research.

### Positive lecturer–student interaction in sociocultural and constructivist context

Research findings indicate that lecturers’ supportive behavior—such as acknowledging student contributions, providing dialogic feedback, and asking provocative questions—plays a crucial role in enhancing self-confidence and critical thinking capacity. As one participant stated, “Such support is very meaningful, especially in developing our confidence during discussions.” This statement aligns with the findings of Che Ahmad et al., (2017), who emphasized that positive classroom interactions strengthen students’ sense of comfort and active participation, two essential prerequisites for higher-order thinking.

From a social constructivist perspective, lecturer–student interactions serve as scaffolding that enables students to internalize analytical processes through dialogue and meaning negotiation ( Wan Hussin et al., 2019b). Hagenauer and Volet (2014) also demonstrated that lecturers’ responsiveness and warmth strengthen students’ motivation, cognitive engagement, and the development of reflective skills. The findings of this study support this argument: students stated that prompting questions, gradual guidance, and verbal appreciation helped them formulate arguments, evaluate information, and reflect more thoughtfully on their academic positions.

However, the effectiveness of these interactions cannot be separated from the Indonesian cultural context. A culture of politeness, respect for hierarchy, and a tendency to avoid confrontation often foster more cautious communication patterns. Some students admitted to feeling hesitant to express opinions or ask questions for fear of being perceived as impolite or misinterpreting the lecturer’s instructions. This phenomenon is consistent with Chung (2021) findings, which demonstrate that silent participation in Southeast Asian countries is often not a form of passivity but rather a strategy for maintaining social harmony and the stability of interpersonal relationships.

Translating this context into the Private Higher Education (PTS) environment adds further significance. Wulandari and Jager (2018) found that small class sizes in PTS create a space for closer and more personal interactions, allowing for more intensive lecturer-student relationships. The findings of this study confirm that these characteristics provide opportunities for lecturers to play a stronger role in mediating learning, guiding the reasoning process, and strengthening students’ courage to participate in discussions.

These findings also resonate with international literature on teacher immediacy. Shoaib, (2023) emphasized that a combination of warm verbal and nonverbal behaviors—such as praise, positive expressions, and supportive gestures—creates a learning climate conducive to students’ academic courage. This finding is supported by Yuan (2024), who demonstrated that teacher immediacy significantly contributes to academic engagement by fostering a strong rapport. In the context of this study, students in private universities consistently rated lecturers’ accessibility, prompt responses, and clear communication as factors that strengthened their participation and courage to think critically.

From a psychological perspective, positive interactions also impact the affective aspects that underlie cognitive abilities. Terblanche et al. (2021) found that lecturers’ emotional support contributed to increased self-esteem, which in turn improved learning initiative, academic participation, and cognitive performance. In this study, students reported that lecturers’ empathy and openness not only fostered interpersonal comfort but also strengthened their self-perceptions as individuals capable of critical argumentation and analysis.

Overall, the findings of this study indicate that positive lecturer-student interactions are not merely interpersonal relationships, but rather pedagogical mechanisms that mediate knowledge construction, foster academic courage, and strengthen critical thinking skills within the sociocultural context of Indonesia and private higher education institutions. Thus, the quality of lecturer-student interactions is a key foundation for meaningful learning, emphasizing dialogue, reflection, and the development of higher-order thinking skills.

### Collaborative peer interaction and the social construction of meaning

The findings of this study indicate that interaction among students through group discussions, collaborative work, and idea exchange is a key catalyst in the development of critical thinking skills. Students acknowledged that “Through tasks and group discussions, we communicate with one another,” which emphasizes collaboration as a dialogic space that broadens perspectives and deepens understanding. Within a sociocultural framework, this pattern reflects Vygotsky’s idea that knowledge construction occurs first in the social realm before being internalized into more complex individual thinking skills.

Consistently, previous studies ( Bellaera et al., 2021; Stigmar, 2016) show that dialogue-based activities help students negotiate meaning, assess the validity of arguments, and formulate more informed interpretations. Collaboration plays a role not only in cognitive processing but also in building motivation, self-confidence, and social engagement. Patra et al. (2022), for example, found that group discussions improved social skills and self-confidence, findings strongly consistent with those reported by students in this study. Collaboration also enriches conceptual understanding by opening access to diverse perspectives, as demonstrated by research by Blau et al. (2020); Puig et al. (2020); and Santoso, (2019).

Furthermore, collaborative learning has been shown to contribute not only to cognitive competence but also to the development of affective and social competencies such as intrinsic motivation, academic empathy, and self-control ( Mendo-Lázaro et al., 2022). In the context of this study, group work becomes a social arena where students develop a sense of agency, intellectual courage, and sensitivity to diverse perspectives—three essential foundations for developing critical thinking skills.

Research findings also show that peer interaction mechanisms significantly influence the quality of critical thinking during the knowledge-building process. Jiang et al. (2023) provided evidence that knowledge-building-based learning communities—through peer assessment, constructive criticism, and the use of counterarguments—resulted in significant improvements in critical thinking skills. A similar pattern was seen in the findings of Xia and Xu (2024), who showed that peer facilitation triggered critical thinking episodes, particularly at the Analyze and Evaluate levels, through a three-layered pattern: bonding, engaging, and monitoring. Meanwhile, a more comprehensive meta-analysis by Xu et al. (2023) concluded that collaborative problem-solving significantly improves critical thinking skills, particularly when the learning design provides a clear group structure, adequate scaffolding, and sufficient intervention duration.

The argumentative dimension of collaboration is also evident in research by Larrain et al., (2019), who found that the active production of counterarguments predicts long-term improvements in understanding. In addition to argumentation mechanisms, diverse voices within a group are a critical element for enriching social reasoning. Lin et al. (2019) emphasized that diverse perspectives enable students to develop more complex reasoning and coordinate knowledge more systematically.

Overall, these findings confirm that collaboration is not merely a task-sharing activity but an epistemic space where students construct, challenge, and revise their understanding through meaningful social interactions. Thus, peer interaction serves as a pedagogical mechanism that strengthens knowledge construction, expands analytical capacity, and fosters critical thinking competencies in a dialogic and inclusive learning environment.

### Interactional barriers: imbalance of support, participation inequity, and cultural constraints

Although the lecturer-student interactions in this study were generally supportive, in-depth analysis revealed several interactional barriers that could hinder the development of critical thinking skills. These barriers primarily relate to imbalances in instructional support, unequal participation in group discussions, and cultural constraints that affect freedom of expression. These three aspects are not isolated but intertwined, forming complex classroom dynamics.

First, students reported variations in the quality and consistency of lecturer support. Some students received in-depth and dialogic feedback, while others reported receiving only brief, general, or inconsistent responses. This imbalance limited students’ opportunities to refine their arguments and identify conceptual errors. These findings align with Van der Kleij & Lipnevich (2021), who asserted that feedback quality is a critical determinant in the development of higher-order reasoning. When instructional support is uneven, students are at a disadvantage in developing the evaluative and reflective skills essential for critical thinking.

Second, unequal participation in group discussions emerged as a significant issue. Several students acknowledged that the dominance of specific individuals inhibited the emergence of alternative perspectives and reduced the distribution of speaking opportunities. This inequality not only narrowed the dialogic space but also created a cognitive opportunity gap—an imbalance in opportunities to test ideas and receive intellectual challenges from peers. This view is consistent with Parker-Shandal (2023), who emphasized the importance of an inclusive discussion climate to avoid reproducing inequalities in academic participation. Furthermore, this phenomenon is intertwined with the pattern of silent participation described by Chung (2021), in which students chose silence as a strategy to maintain harmony or avoid evaluative risks.

Third, limited freedom of expression emerged as the most obvious barrier impacting the development of critical thinking. Students noted that they were cautious—even hesitant—to express dissenting views, especially if they conflicted with the majority or the professor’s position. Fear of being wrong, fear of negative judgment, and hierarchical norms reinforced patterns of self-censorship. This aligns with the findings of Wijayanti et al. (2023) and Sari et al. (2023) which show that norms of politeness and structural power relations strongly influence the dynamics of academic dialogue in Indonesia. Svendby (2024) critique—that education should not resemble a “factory” that suppresses individual expression—is relevant to interpreting how structural barriers can reduce the space for developing students’ critical voices.

These limitations have direct implications for the quality of the exchange of ideas. When the space for presenting alternative arguments is narrowed, students’ opportunities to evaluate and improve their reasoning are also limited. This is consistent with research by Lasfeto and Ulfa (2020), which shows that the fear of being wrong inhibits students’ courage to think critically. Conversely, a study by Espasa et al. (2022) demonstrated that supportive dialogue—even through digital media such as video feedback—can strengthen connectedness and increase student participation in evaluative and reflective activities.

When viewed from an international perspective, this pattern of barriers aligns with findings that depict student silence as a complex social phenomenon rather than simply individual passivity. Wang et al. (2022) emphasized that L2 (second language) students’ silence is often misunderstood, yet it is, in part, a legitimate and strategic form of participation. In previous work, Wang and Moskal (2019) demonstrated that this silence is rooted in interaction norms, a sense of membership in a community of practice, and the process of adapting to a new academic culture. This perspective is reinforced by Chung (2021), who asserts that Southeast Asian students often engage in “quietly attentive participation” to maintain harmony and manage social risk.

In the broader Southeast Asian context, Le (2024) asserted that academic hierarchies and the authoritative figures of lecturers create a psychological distance that limits students’ courage to ask questions or challenge ideas. This finding is highly relevant to research on private universities, where students consistently express high regard for lecturers who are accessible, responsive, and demonstrate verbal and nonverbal immediacy. Lecturers’ responsiveness and warmth have been shown to reduce hierarchical distance, increase self-confidence, and open up space for more equal critical participation.

Therefore, the interactional barriers identified in this study need to be understood as social phenomena formed by the interaction between linguistic competence, cultural norms, power structures, and the quality of lecturer-student communication. When lecturers create an egalitarian, empathetic, and responsive learning environment, these barriers can be transformed into opportunities to build a more inclusive discursive space conducive to the development of critical thinking skills.

The finding that supportive interactions with lecturers and collaborative dynamics among students directly strengthen self-confidence, engagement, and critical thinking underscores the need for pedagogical training that emphasizes dialogic communication, consistent feedback, and the facilitation of inclusive discussions. Furthermore, the identification of barriers to participation—including group dominance, hierarchical norms, and silent participation—implies the importance of learning designs that create safe spaces, equitable discussion structures, and diverse formats for student expression in private universities.

This study’s limitations include its small sample size at a single private university and its reliance on subjective student reports. Therefore, the findings cannot be broadly generalized to different institutional contexts and academic cultures. Future research should expand the context and methods by incorporating classroom observations, cross-institutional comparisons, and in-depth exploration of the mechanisms of silent participation to understand how cultural factors and power relations influence student critical engagement.

## Conclusions

This research demonstrates that learning interactions play a central role in shaping students’ critical thinking skills, particularly in Indonesian private universities. From the outset, this study examines the urgency of strengthening 21st-century skills and the institutional challenges faced by private university students, including reliance on interpersonal relationships, variability in teaching quality, and the influence of a hierarchical academic culture. Within this context, this research highlights that lecturer-student interactions and student-to-student collaboration are two key arenas where critical thinking skills are developed or hindered.

Overall, this research concludes that students’ critical thinking skills develop when learning interactions are supportive, equitable, dialogic, and allow for a diversity of voices. Conversely, unequal interactions, hierarchical communication, and a culture of silence can limit intellectual participation and narrow the space for critical reflection. Therefore, strengthening the quality of lecturer-student interactions and the governance of collaborative discussions are important strategies for universities—particularly private universities—to build a learning environment that truly supports students’ critical, creative, and reflective development.

The thematic structure of findings regarding lecturer–student interaction and collaborative learning is summarized in Figure 1, which illustrates the main categories emerging from the interviews.

**Figure 1.**
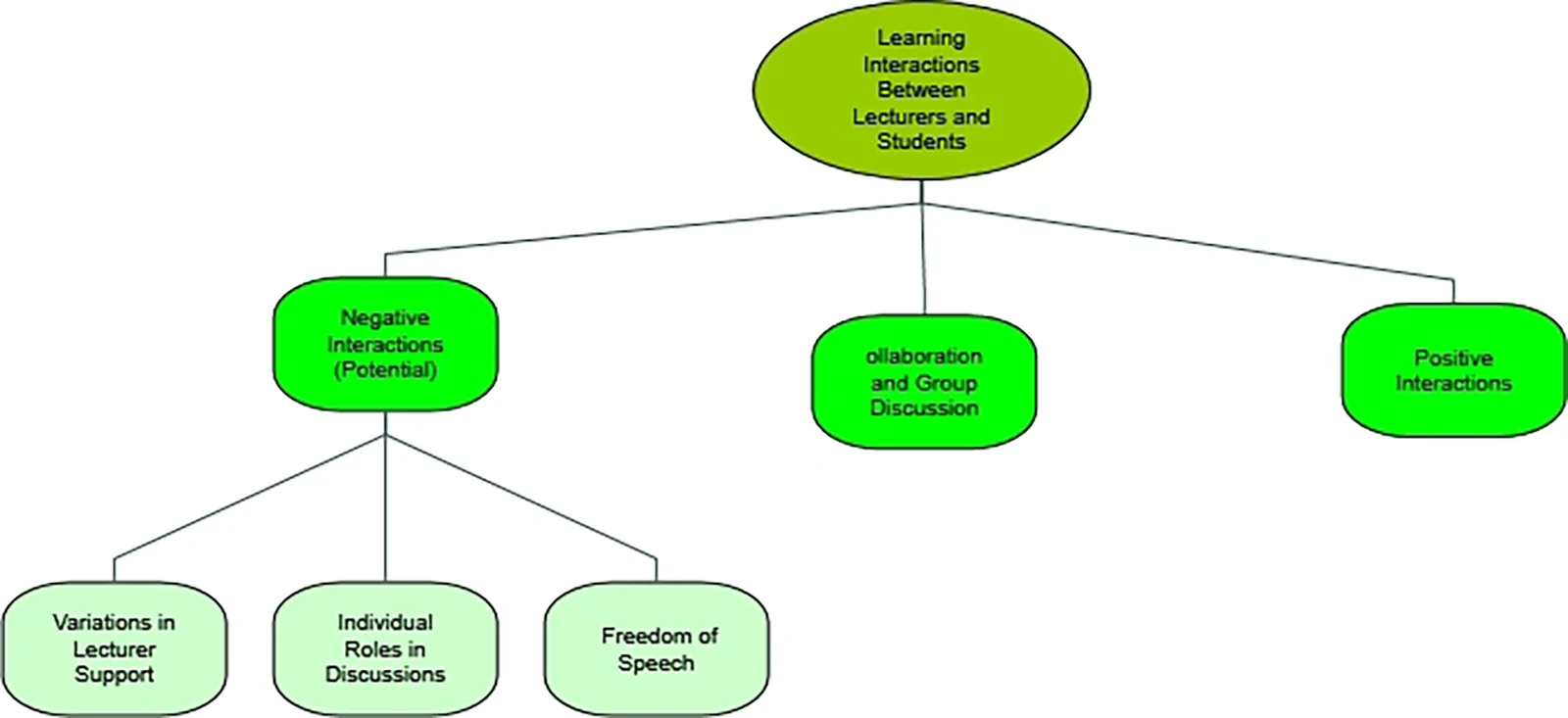
Findings in the learning interactions between lecturers and students.

### Ethical considerations

Ethical approval for this study was granted by the Research and Community Service Department of Universitas Djuanda, Bogor, Indonesia (Certificate No. 341/LPPM/K-X/X/2024, issued on February 8th, 2024). Written informed consent was obtained from all participants in accordance with approval document 01/K-X/IX/2024, dated January 24th, 2024.

## Data Availability

Underlying and extended data supporting the findings of this study are available in Zenodo at: The Role of Lecturer–Student Interaction in Developing Critical Thinking Skills: Insights from a Private University in Indonesia. https://doi.org/10.5281/zenodo.17927148 ( Prasetyo et al., 2025). This repository contains:
• Interview transcripts (English translation) • Coding extract • Codebook Data are available under the terms of the Creative Commons Attribution 4.0 International license (CC-BY 4.0).
