# Supplementary material for: The Role of Lecturer–Student Interaction in Developing Critical Thinking Skills: Insights from a Private University in Indonesia
Source: F1000Res. 2025 Dec 26;14:1455. [Version 1] doi: 10.12688/f1000research.173679.1 (PMC13736058; doi:10.12688/f1000research.173679.1)
Supplement: Peer review history

# Reviewer report: Reviewer response for version 1

**DOI:** 10.5256/f1000research.191517.r448359

**Published:** 2026-02-05

**Authors:** Fahmi Fahmi

**Affiliations:** 1 Universitas Lambung Mangkurat, Banjarmasin, South Kalimantan, Indonesia

Some notes to note

1. Fix typos: "LecturerStudent" → "lecturer-student"; remove "version 1 peer review 1 not approved".
2. Translate all Indonesian phrases (e.g., "Ketika ditanya...") to full English.
3. Standardize hyphenation/spacing; proofread entire text with Grammarly/APA style checker.
4. Use consistent participant labels: "Participant 1" instead of "Subject 1".
5. Delete duplicate paragraphs in Introduction (Vygotsky, Southeast Asian cultural norms).

Methodology

1. Add FGD details: participant count, duration, full triangulation protocol.
2. Include sampling criteria: inclusion/exclusion, major/semester variation.
3. Provide 2-3 sample interview questions and simple codebook excerpt.
4. Clarify analysis: specify number of major themes (not vague "one major themes").

Results & Analysis

1. Create summary table: columns for Theme, Quote Frequency, Representative Example.
2. Link findings to critical thinking definition (e.g., Facione's rubric).
3. Reduce repeated participant quotes; limit to 1-2 per theme.

References

1. Complete all DOIs/links; ensure consistent APA 7th formatting.

Is the work clearly and accurately presented and does it cite the current literature?

Yes

If applicable, is the statistical analysis and its interpretation appropriate?

Partly

Are all the source data underlying the results available to ensure full reproducibility?

Yes

Is the study design appropriate and is the work technically sound?

Partly

Are the conclusions drawn adequately supported by the results?

Yes

Are sufficient details of methods and analysis provided to allow replication by others?

Yes

Reviewer Expertise:

Education, learning innovation, critical thinking

I confirm that I have read this submission and believe that I have an appropriate level of expertise to confirm that it is of an acceptable scientific standard, however I have significant reservations, as outlined above.

# Reviewer report: Reviewer response for version 1

**DOI:** 10.5256/f1000research.191517.r448353

**Published:** 2026-02-03

**Authors:** Rita Pramujiyanti Khotimah

**Affiliations:** 1 Universitas Muhammadiyah Surakarta, Surakarta, Indonesia

The conclusion presented in the abstract does not adequately explain the barriers influencing the development of students’ critical thinking. In the introduction, the rationale for focusing specifically on critical thinking—rather than other related skills such as communication, collaboration, or reasoning—is not sufficiently justified. Additionally, the research gap that underscores the significance of the study’s objectives is not clearly articulated.

 Regarding the research subjects, the manuscript does not explain the subject selection method. In particular, the criteria and rationale for selecting twelve undergraduate students (seven females and five males) are unclear, including the relevance of gender distribution to the study.

 In the results section, the manuscript is not written entirely in English, and the data presentation lacks clarity and engagement. Several themes—such as positive interactions, collaboration and group discussion, potential negative interactions, variations in lecturer support, individual roles in discussions, and freedom of speech—are introduced abruptly without sufficient explanation or justification. Moreover, the results do not adequately describe the forms of interaction, the quality of pedagogical support, or the barriers that affect the development of students’ critical thinking.

 Finally, the conclusion does not sufficiently address or answer the stated research purposes.

Is the work clearly and accurately presented and does it cite the current literature?

Partly

If applicable, is the statistical analysis and its interpretation appropriate?

Not applicable

Are all the source data underlying the results available to ensure full reproducibility?

Partly

Is the study design appropriate and is the work technically sound?

Partly

Are the conclusions drawn adequately supported by the results?

Partly

Are sufficient details of methods and analysis provided to allow replication by others?

Partly

Reviewer Expertise:

STEM education, Teaching and learning

I confirm that I have read this submission and believe that I have an appropriate level of expertise to state that I do not consider it to be of an acceptable scientific standard, for reasons outlined above.

# Response

**Published:** 2026-02-03

**Authors:** Teguh Prasetyo

**Affiliations:** Universitas Djuanda, Bogor, West Java, Indonesia

We sincerely thank the reviewer for the constructive and detailed feedback. We acknowledge that substantial revisions are required and we are currently revising the manuscript to address all comments, including strengthening the abstract, introduction, methodology, results, and conclusion.
